# Prognostic implications of the BRAF-V600^E^ mutation in papillary thyroid carcinoma based on a new cut-off age stratification

**DOI:** 10.3892/ol.2019.11132

**Published:** 2019-11-21

**Authors:** Xiaoxiong Gan, Fei Shen, Xingyan Deng, Jianhua Feng, Jiabao Lu, Wensong Cai, Lina Peng, Weipeng Zheng, Weijia Wang, Peidan Huang, Zhen Chen, Mengli Guo, Bo Xu

**Affiliations:** 1 Department of Thyroid Surgery, Guangzhou First People's Hospital, Guangzhou Medical University, Guangzhou, Guangdong 510180, P.R. China; 2 Department of Colorectal and Anal Surgery, Guangzhou Digestive Disease Center, Guangzhou First People's Hospital, Guangzhou Medical University, Guangzhou, Guangdong 510180, P.R. China; 3 Department of General Surgery, Shenzhen Hospital, Southern Medical University, Shenzhen, Guangdong 518101, P.R. China; 4 Department of Thyroid Surgery, Guangzhou First People's Hospital, Second Affiliated Hospital of South China University of Technology, Guangzhou, Guangdong 510180, P.R. China

**Keywords:** papillary thyroid carcinoma, BRAF mutation, prognosis, recurrence

## Abstract

The BRAF-V600^E^ mutation is the most common and specific oncogenic event known in papillary thyroid carcinoma (PTC). However, it remains controversial whether there is an association between the BRAF-V600^E^ mutation and clinicopathologically aggressive characteristics of PTC. The purpose of the present retrospective study was to investigate the significance of the BRAF-V600^E^ mutation in predicting prognostic and aggressive clinicopathological characteristics according to a new age-based stratification. Clinical data and the BRAF-V600^E^ mutation status of 475 patients with PTC were downloaded from The Cancer Genome Atlas database. The association between BRAF-V600^E^ status and clinicopathological characteristics was analyzed by χ^2^ test or Fisher's exact test. Recurrence-free survival rate (RFS) was analyzed using the Kaplan-Meier method. Aggressive clinicopathological factors associated with recurrence were analyzed by Cox multivariate regression. This study was conducted on 219 cases of patients with PTC with a known BRAF-V600^E^ mutational status. In the ≥55 years age group, BRAF-V600^E^ was found to be significantly associated with aggressive PTC characteristics, including tumor size, PTC subtype, radioactive iodine (RAI) dose, follow-up time, recurrence, recurrence risk stage, advanced T stage, advanced N stage and American Joint Committee on Cancer (III/IV) stage (all P<0.05). RFS was analyzed by the log-rank test and exhibited statistically significant differences in the ≥55 years group (P=0.041), but there was no significant difference in the <55 group (P=0.757), according to the BRAF-V600^E^ mutation status. The BRAF-V600^E^ gene was excluded from the recurrence Cox multivariate regression model. The BRAF-V600^E^ mutation was found to better predict aggressive and recurrent PTC based on age stratification with the cut-off age of 55 years. The synergistic interaction between BRAF-V600^E^ mutation and the new age stratification may help clinicians reach the optimal decision in terms of surgical approach and the extent of surgery.

## Introduction

Thyroid cancer is the most common endocrine malignancy, and its incidence has rapidly increased globally over the past few decades. The incidence rate of thyroid cancer tripled from 4.9 to 14.3 per 100,000 individuals between 1975 and 2009 (1). Papillary thyroid carcinoma (PTC) is the most frequent type of thyroid cancer, accounting for 80–90% of all thyroid malignancies (2), and conventional PTC is the main histological variant (3).

The BRAF-V600^E^ mutation gene is the most frequent oncogenic genetic alteration in PTC, accounting for approximately 45% of PTC cases, and has been shown to be associated with a poor prognosis of PTC (4). This mutation is associated with aggressive tumor behavior, disease recurrence and disease-specific mortality in PTC (5–7). Numerous studies have documented the oncogenic molecular mechanisms of BRAF-V600^E^ driving the aggressiveness of PTC (8,9). The American Joint Committee on Cancer (AJCC)/Union for International Cancer Control reconvened in 2016 to update the tumor node metastasis (TNM) staging system and published its 8th edition, in which significant changes were introduced regarding thyroid cancer (10). The age factor is a major change in the revised 8th edition of AJCC, as the age of 55 years has been introduced as the cut-off point demarcating the age-related prognostic risk of thyroid cancer, and patients aged >55 years appear to have a worse prognosis compared with patients younger than 55 years (10). A large multicenter study by Xing *et al* (5) demonstrated that the BRAF-V600^E^ mutation was significantly associated with the risk of recurrence in PTC. Based on these data, it appears that the BRAF-V600^E^ status in isolation is not sufficient to substantially contribute to risk stratification in the majority of the patients. In addition, an incremental improvement in risk stratification may be achieved if the BRAF-V600^E^ mutational status is considered in the context of other standard clinicopathological risk factors (11).

Therefore, synergistic interaction between BRAF-V600^E^ mutation and age stratification may better predict the prognosis of PTC. However, it was previously reported that the BRAF-V600^E^ mutational status was not an independent prognostic factor when patients were grouped by age into two categories (<45 and ≥45 years) and into three categories (<35, 35–60 and ≥60 years) (12). The aim of the present study was to investigate whether the synergistic interaction between BRAF-V600^E^ mutational status and the new age stratification with a cut-off age of 55 years is more efficient in predicting the prognosis of PTC.

## Materials and methods

### 

#### Data source

The Cancer Genome Atlas (TCGA) Data Portal (https://tcga-data.nci.nih.gov) is the result of a collaboration between the National Cancer Institute and the National Human Genome Research Institute (13). A comprehensive multi-dimensional map of the key genomic changes in 33 types of cancer has been generated. High-quality tumor and matched normal samples from 507 patients with thyroid cancer were collected and identified via the TCGA database, of which 475 patients had complete clinicopathological data.

#### Patient grouping and definition

A total of 475 patients were divided into two groups according to the new cut-off age, namely the <55 years and the ≥55 years age groups. Lymph node positivity was defined as a count of positive lymph nodes >5. Tumors were staged according to the TNM staging system recommended by the 8th edition of AJCC (10). Based on the 2015 American Thyroid Association (ATA) Management Guidelines (11), patients with gross extrathyroidal extension (ETE), incomplete tumor resection, distant metastases or lymph nodes >3 cm were classified into the high risk of recurrence group; those with aggressive histological characteristics, minor ETE, vascular invasion, or >5 involved lymph nodes (0.2–3 cm) were classified into the intermediate risk of recurrence group; and those with intrathyroidal differentiated thyroid cancer (DTC) (11) and ≤5 lymph node micrometastases (*<*0.2 cm) were considered as the low risk of recurrence group.

#### General information

In the present study, gene-level gene expression data from mRNA-seq, BRAF-V600^E^ mutation data and clinicopathological information of 475 patients with PTC were extracted from TCGA up until September 26, 2018. A total of 475 patients diagnosed with PTC, including 352 women (74.1%) and 123 men (25.9%), were investigated. The median age of the patients was 46 years [interquartile range (IQR), 35–58 years]. Based on the cut-off age of 55 years, the patients were divided into the <55 years and the ≥55 years age groups. Patient age, sex, ethnicity, tumor size, multifocality, lymphocytic thyroiditis, histology, lymph node positivity, ETE, residual tumor, radioactive iodine (RAI) therapy, RAI dose, recurrence follow-up time, mortality follow-up time, TNM stage and AJCC stage (Tables I and II) were compared between the two groups. The overall BRFA-V600^E^ mutation prevalence was 50.3% and the overall PTC recurrence rate was 6.4%.

#### Clustering analysis

Genes that were differentially expressed between positive and negative for BRAF-V600^E^ mutation status in the two age groups were assessed using RStudio software program (http://www.r-project.org). The heat map was generated using ‘pheatmap’ package in ‘R’ software (RStudio version 1.1.463) (14) to visualize the gene expression pattern, with red and green color representing highly expressed and lowly expressed genes, respectively.

#### Statistical analysis

Age, tumor size, and RAI dose are presented as mean ± SD while recurrence and mortality follow-up times are presented as median and quartile ranges Sex, ethnicity, tumor foci, lymphocytic thyroiditis, histology, lymph nodes positivity (>5), ETE, residual tumor, RAI therapy, recurrence, recurrence risk stage, T stage, N stage, M stage and AJCC stage are presented as the frequency. The statistical analysis was performed using SPSS, version 19 (IBM Corp.). The association of BRAF-V600^E^ mutation status and each clinicopathological variable was assessed using the Pearson's χ^2^ test and Fisher's exact test when the number of patient cases was *<*5. The Kaplan-Meier analysis and log-rank test were used to analyze recurrence-free survival (RFS) distribution and to compare the differences between Kaplan-Meier curves for BRAF-V600^E^ status. The odds ratio (OR) was determined by univariate analysis, and ORs with 95% confidence intervals (CIs) were calculated. Multivariate analyses were conducted with the Cox regression analysis method on disease recurrence, and hazard ratios (HRs) with 95% CIs were calculated. All P-values were two-sided, and P<0.05 was considered to indicate statistically significant differences.

## Results

### 

#### Patient demographics

The overall median follow-up time for all patients was 20 months (IQR, 14–38.3 months). The median follow-up time was 21 months (IQR, 14–44 months) in the <55 years group and 19 months (IQR, 12–34 months) in the ≥55 years group.

#### Association between BRAF-V600^E^ mutation status and clinicopathological parameters in the <55 years age group

In this group, of the 318 patients who were diagnosed with PTC [including 249 women (78.3%) and 69 men (21.7%), with a mean age at diagnosis of 38±10 years (range, 15–54 years) and a mean tumor size of 1.7±0.8 cm], 232 patients (51.6%) were positive for BRAF-V600^E^ mutation.

The univariate analyses demonstrated a significant association between the presence of BRAF-V600^E^ and tumor size (P<0.001), multifocality (P=0.036), histology (P<0.001), RAI dose (P<0.001), follow-up time (P=0.001) and recurrence risk stage (P<0.001), whereas there was no significant association with age, sex, ethnicity, lymphocytic thyroiditis, lymph node positivity, ETE, residual tumor, RAI treatment, recurrence, TNM stage and AJCC stage (Table I).

**Figure 1.**
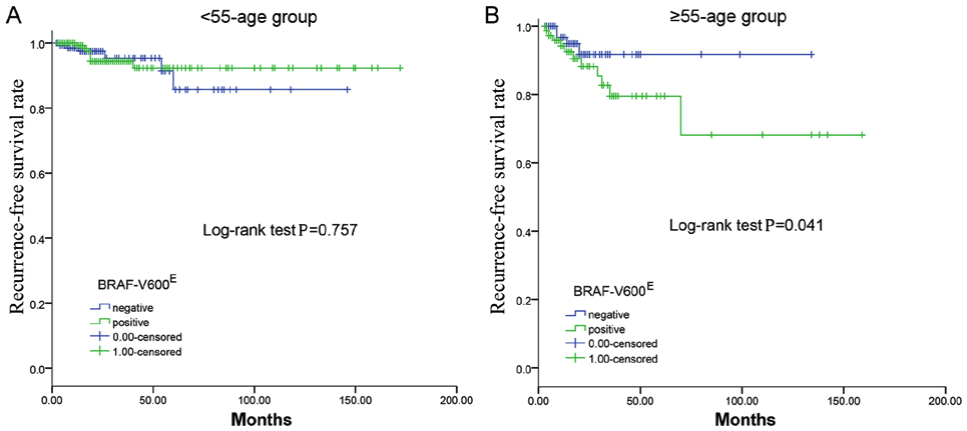
Kaplan-Meier survival analysis of RFS in patients according to BRAF-V600^E^ status. (A) There was no significant difference in survival between the patients with or without the BRAF-V600^E^ mutation in the <55-age group. (B) Patients with the BRAF-V600^E^ mutation had worse RFS compared with patients without the BRAF-V600^E^ mutation in the ≥55-age group. RFS, recurrence-free survival. The 0.00-censored indicates patients who are negative for the BRAF-V600^E^ mutation and the 1.00-censored indicates patients who are positive for the BRAF-V600^E^ mutation.

The median follow-up time was 21 months (IQR, 14–44 months), during which time 13 patients (4.5%) developed recurrence; there were no reported fatalities. The univariate analyses revealed no significant association between the BRAF-V600^E^ mutation and PTC recurrence (P=0.865). Furthermore, the Kaplan-Meier plot for RFS did not reveal a statistically significant difference between the presence and absence of BRAF-V600^E^ (P=0.757; Fig. 1A).

#### Association between BRAF-V600^E^ mutation status and clinicopathological parameters in the ≥55 years age group

The group included a total of 157 patients diagnosed with PTC, including 103 women (65.6%) and 54 men (34.4%). The mean age of the patients was 65±9 years and the mean tumor size was 1.9±1.0 cm. The prevalence rate of the BRAF-V600^E^ mutation was 47.8% (75 cases).

In the ≥55 years age group, the results on univariate analysis demonstrated a significant association between BRAF-V600^E^ mutation and tumor size (P=0.036), histology (P<0.001), RAI therapy dose (P=0.032), recurrence follow-up time (P=0.018), recurrence (P=0.031), mortality follow-up time (P=0.006), recurrence risk stage (P<0.001), advanced T stage (P=0.036), advanced N stage (P=0.036) and advanced AJCC stage (P=0.016) (Table II). There was no significant association between BRAF-V600^E^ and age, sex, ethnicity, multifocality, lymphocytic thyroiditis, lymph node positivity, ETE, residual tumor, RAI therapy, mortality or M stage (all P≥0.05).

During a mean follow-up of 28 months (range, 3–157 months), 15 cases (10.2%) of recurrence were recorded. The presence of BRAF-V600^E^ mutation was associated with lower RFS in the ≥55 year age group (P=0.041; Fig. 1B).

#### Recurrence risk factors

**Figure 2.**
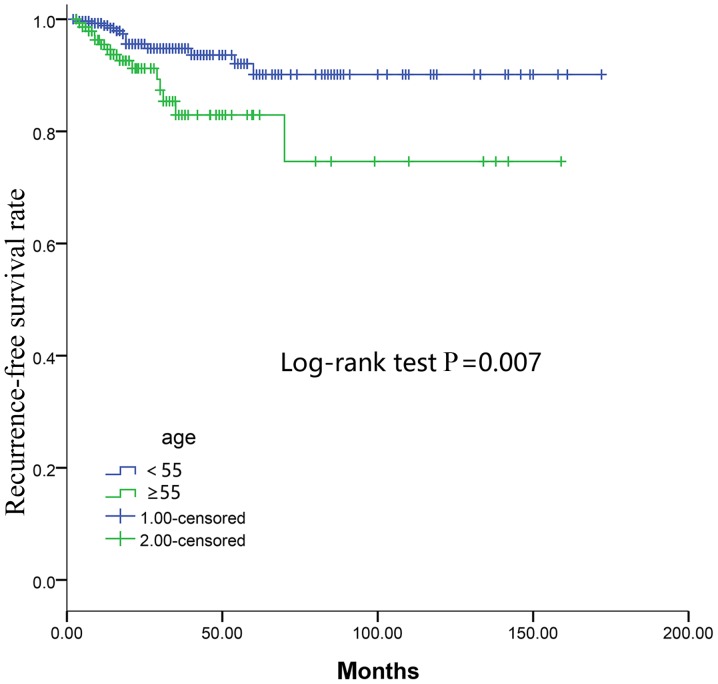
Kaplan-Meier survival analysis of RFS. Patients in the ≥55-age group had worse RFS rates compared with patients in the <55-age group. RFS, recurrence-free survival. The 1.00-censored indicates patients in the <55 age group and the 2.00-censored indicates patients in the ≥55 age group.

Multivariate regression analysis controlling for BRAF-V600^E^, male sex, multifocality, histology, ETE, residual tumor, T stage, N stage, M stage and AJCC stage found that only advanced N stage (P=0.038) and advanced M stage (P=0.028) were independent predictors of recurrence in the <55 age group (Table III). Notably, multivariate analysis demonstrated that male sex (P=0.026), multifocality (P=0.034), N stage (P=0.001) and M stage (P=0.005) were independent predictors of recurrence in the ≥55 age group (Table III). However, the Cox multivariate regression demonstrated that BRAF-V600^E^ was not an independent predictor of recurrence in either of the two groups (P=0.758 and 0.993, respectively; Table III). In addition, the Kaplan-Meier curves of RFS revealed that the ≥55 age group had a lower RFS rate compared with the <55 age group as determined by the log-rank test (P=0.007; Fig. 2).

#### Heat map result by clustering analysis

**Figure 3.**
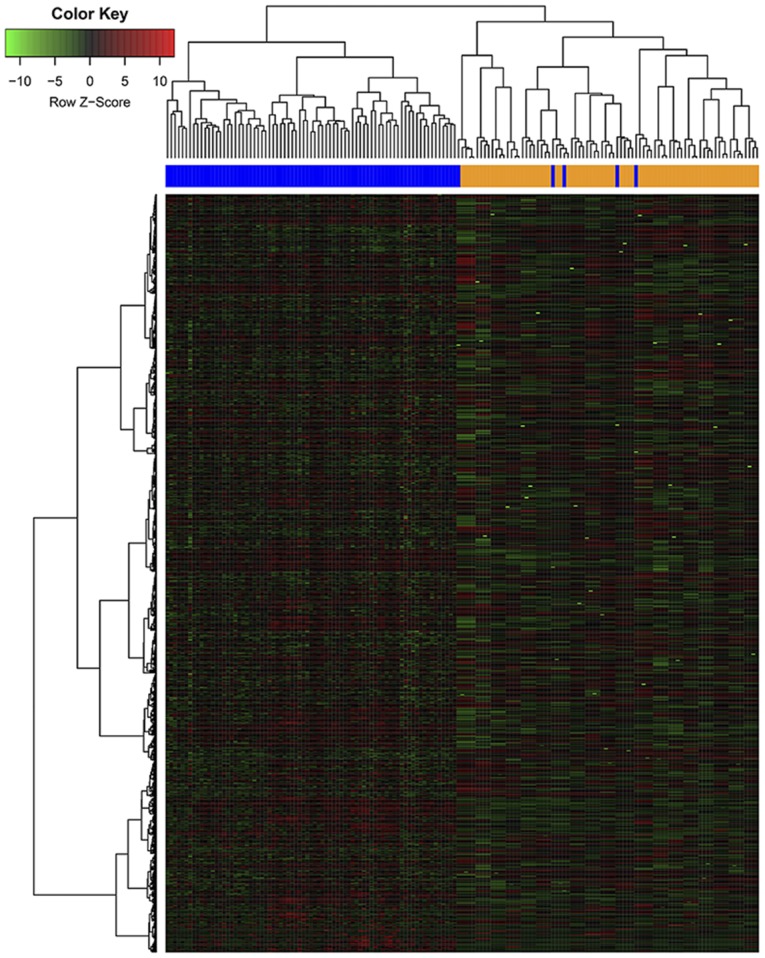
Heat map results of the of BRAF-V600^E^ mutation-positive and -negative cases in the ≥55 years age group. Gene expression pattern according to the BRAF-V600^E^ status. Supervised clustering of PTCs did exhibit a significant clustering effect between BRAF-V600^E^ mutation-positive and -negative status. Each cell in the matrix represents the expression level of a gene feature in an individual pattern. Red or green, high or low expression, respectively, as indicated in the scale bar. PTC, papillary thyroid carcinoma.

Gene variability was then computed using the median absolute deviation. The 3,120 most variable genes were selected. The heat map generated from the gene expression data revealed that there was a significant clustering effect between the positivity and negativity for BRAF-V600^E^ mutation in the ≥55 year age group (Fig. 3). By contrast, the gene expression data demonstrated that there was no significant difference between the two groups in the <55 year age group.

## Discussion

PTC is a well-differentiated papillary carcinoma with a relatively low mortality rate among thyroid cancers (14). However, the rate of disease recurrence or persistence is high, up to 30% (15,16). The BRAF-V600^E^ mutation has been reported as a prognostic molecular marker in PTC (8,17,18). However, the prevalence of the BRAF-V600^E^ mutation in PTC ranges between 30 and 80% (19). Therefore, this molecular marker is of limited value for clinical decision-making in the majority of PTC cases. For thyroid cancer, the recent 8th edition of the AJCC staging system strongly emphasizes the overall age-related risk, with a cut-off age at 55 years (10). In addition, several studies have demonstrated an association of BRAF-V600^E^ with older age and poor clinical outcomes, including PTC recurrence and PTC-specific mortality (5,7). The present study investigated the hypothesis that BRAF-V600^E^ may better predict PTC aggressiveness and recurrence based on this age stratification.

This study demonstrated no significant difference between the presence of BRAF-V600^E^ mutation and ethnicity, in the ≥55-age group and the <55-age group. Therefore, we believe that the BRAF-V600^E^ mutation is not significantly heterogeneous in different ethnicities. By categorizing patients in the aforementioned two age groups the effectiveness of BRAF-V600^E^ in predicting aggressiveness and recurrence of PTC in terms of the cut-off age at 55 years improved. Furthermore, the heat map revealed significant clustering between the positive and negative BRAF-V600^E^ cases in the ≥55 age group. Similar to the results reported by Xing *et al* (5), in the ≥55 group, the presence of the BRAF-V600^E^ mutation was significantly associated with tumor size, histology, RAI dose, lymph node metastasis (LNM), advanced AJCC stage (III/IV) and tumor recurrence, which are the major factors associated with a worse prognosis of PTC (11). By contrast, in the <55 age group, the prognostic implications of the BRAF-V600^E^ mutation in PTC were limited.

In the present study, the RFS distribution suggested that the ≥55 group exhibited a lower survival rate compared with the <55 group, and the latter group had a better prognosis. In addition, it was demonstrated that BRAF-V600^E^ is useful for predicting prognosis based on age stratification with the cut-off at 55 years. The molecular mechanism underlying the age-dependent effect of the BRAF-V600^E^ mutation on the prognosis of patients with PTC remains to be defined (20). It is possible that certain age-related genes, such as immune response-associated genes (21), may cooperate with BRAF-V600^E^ in conferring poor prognosis, as BRAF-V600^E^ was shown to be associated with abnormal immune response in human cancers, including PTC (22,23). The present study further demonstrated that the presence of the BRAF-V600^E^ mutation was associated with a lower survival rate on RFS analysis in the ≥55 age group. However, in the <55 age group, there was no significant difference in survival between the presence and absence of the BRAF-V600^E^ mutation. Therefore, BRAF-V600^E^ may better predict the prognosis and recurrence of PTC based on the 55 year age stratification.

In addition, this study demonstrated that the synergistic interaction of BRAF-V600^E^ and age stratification was of greater assistance for clinicians in terms of optimal decision-making regarding surgical approach and the extent of surgery. It has been reported that central LNM is the most common cause of disease recurrence in PTC (24,25). However, prophylactic central lymph node dissection is not routinely recommended based on the currently published guideline (11). The results of the present study demonstrated that BRAF-V600^E^ was significantly associated with LNM in the ≥55 age group, and positive BRAF-V600^E^ mutation status was associated with a higher risk of LNM compared with the negative mutation status. This study confirmed the association between the BRAF-V600^E^ mutation and a higher risk of LNM in the ≥55 age group. By contrast, in the <55 age group, there was no significant association between BRAF-V600^E^ and LNM. Whether the presence of BRAF-V600^E^ with regional LNM exert a synergistic effect on increasing the rate of locoregional disease recurrence remains elusive. Further RFS analysis demonstrated that there was no significant difference between positive and negative BRAF-V600^E^ mutation status in the LNM subgroup in the two age groups. Therefore, prophylactic central neck dissection may be performed in positive BRAF-V600^E^ mutation cases, particularly in the ≥55 years age group. However, the synergistic interaction between BRAF-V600^E^ and LNM is unclear, and patients with PTC who are BRAF-V600^E^-positive with LNM may not require more aggressive treatment compared with patients with PTC who are BRAF-V600^E^-negative with LNM.

Although RAI is routinely recommended by the 2015 ATA guidelines for patients with intermediate- to high-risk DTC, there is currently no consensus regarding the dose required for ablation (11). There may be an association between the BRAF-V600^E^ mutation and the expression of certain genes, including sodium/iodide symporter, thyroid-stimulating hormone receptor, thyroperoxidase, thyroglobulin, and pendrin (26). The sodium/iodide symporter gene is involved in RAI metabolism, therefore mutations could result in impaired sodium/iodide symporter expression, and also a decrease in iodide-metabolizing gene expression of thyrotropin receptor, thyroglobulin and thyroperoxidase (27). This may be a possible explanation for RAI therapy failure and recurrence of PTC (28). Although tumors with the BRAF-V600^E^ mutation tend to be RAI-resistant, that knowledge, to the best of our knowledge, has yet to affect decision-making regarding RAI therapy. The results of the present study revealed no significant association between BRAF-V600^E^ and RAI therapy in the <55 and ≥55 age groups. However, the presence of BRAF-V600^E^ was associated with higher RAI therapy dose in the ≥55 age group. A large multicenter study reported that the association between BRAF-V600^E^-positive mutation and high-dose RAI therapy is controversial (5). Further research conducted by the present study revealed no significant difference between high- and low-dose RAI on RFS analysis in a low- to intermediate-risk group (based on the 2015 ATA risk stratification system). Therefore, it is uncertain whether higher-dose RAI therapy is required for patients with PTC, who harbor the BRAF-V600^E^ mutation. However, larger samples and cohort studies are required to confirm whether older patients with the BRAF-V600^E^ mutation require high-dose RAI therapy to improve prognosis.

In addition, the presence of BRAF-V600^E^ was not found to be an independent prognostic factor for predicting recurrent disease on univariate and multivariate analyses, when patients were grouped into <45 and ≥45 years age groups (12). Although BRAF-V600^E^ had no independent impact on RFS, the BRAF-V600^E^ mutation better predicted aggressive clinicopathological characteristics and PTC recurrence based on age stratification using 55 years as the cut-off.

There were several limitations to the present study. First, the sample size was relatively small, with 475 cases downloaded from TCGA. Second, not all of the clinicopathological data was complete therefore some data is missing. Third, this study was a retrospective analysis using TCGA data, and prospective clinical trials are required to provide more reliable results. Fourth, information associated with the extent of surgery and thyrotropin suppression is not recorded by TCGA. Finally, the sex ratio of women to men is 352 to 123, consistent with the ratio of epidemiology (3.1:1) (1). However, in the <55 age group, gender distribution was unequal at 249 women and 69 male, which may cause bias.

In summary, the BRAF-V600^E^ mutation in PTC better predicts aggressive and recurrent disease based on stratification by 55 years of age. The synergistic interaction between the BRAF-V600^E^ mutation and age stratification with a cut-off of 55 years may be more helpful for clinicians and facilitate optimal decision-making regarding surgical approach and the extent of surgery.

## Figures and Tables

**Table I.** Univariate analyses of association between BRAF-V600^E^ mutation status and clinicopathological parameters in <55-age group.

		BRAF-V600^E^			
				
Patients' parameters	Total	Mutation	Wild-type	Odds ratio (95% CI)	P-value
Age, years (mean ± standard deviation)	38±10	39±10	37±10	NA	0.083
Sex, n					
Female	249	128	121	1	0.910
Male	69	36	33	1.031 (0.605–1.759)	
Ethnicity category, n					
Caucasian	207	116	91	1	0.742
Asian	38	20	18	0.872 (0.436–1.744)	
Black	15	7	8	0.686 (0.240–1.963)	
Tumor size, cm (mean ± standard deviation)	1.7±0.8	1.6±0.9	1.7±0.8	NA	<0.001a
Tumor foci, n					
Unifocality	285	36	249	1	0.036a
Multifocality	33	0	33	1.133 (1.085–1.182)	
Lymphocytic thyroiditis, n					
No	240	119	120	1	0.099
Yes	45	29	17	1.720 (0.898–3.296)	
Histology, n					
CPTC	237	142	95	1	<0.001a
FVPTC	63	9	54	0.112 (0.053–0.237)	
TCPTC	18	13	5	1.739 (0.600–5.039)	
Lymph nodes positivity, n (>5)					
No	190	102	88	1	0.618
Yes	60	30	30	0.863 (0.483–1.542)	
ETE, n (gross)					
No	307	160	147	1	1.000b
Yes	2	1	1	0.919 (0.057–14.822)	
Residual tumor, n					
No	253	137	116	1	0.098
Yes	29	11	18	0.517 (0.235–1.140)	
RAI therapy, n					
No	123	62	61	1	0.771
Yes	163	85	78	1.072 (0.671–1.713)	
RAI dose, mCi (mean ± standard deviation)	121±59	120±50	122±68	NA	<0.001a
Recurrence follow-up, months	21 (14–44)	26 (16–50)	18 (12–39)	NA	0.001a
Recurrence, n					
No	278	143	135	1	0.865
Yes	13	7	6	1.101 (0.361–3.360)	
Mortality follow-up, months	21 (13–44)	25 (15-51.25)	18 (12-39.5)	NA	0.001a
Mortality, n					
No	318	164	154	1	NA
Yes	0	0	0	NA	
Recurrence risk stage, n					
Low	108	26	82	1	<0.001a
Intermediate	163	124	39	10.028 (5.675–17.719)	
High	33	13	20	2.050 (0.898–4.682)	
T stage, nc					
1	221	114	107	1	0.981
2	69	36	33	1.024 (0.596–1.759)	
3	28	14	14	0.939 (0.428–2.061)	
4	0	NA	NA	NA	
N stage nc					
0	133	62	71	1	0.105
1a	68	42	26	1.850 (1.019–3.357)	
1b	45	21	24	1.002 (0.509–1.973)	
M stage, nc					
0	178	96	82	1	1.000b
1	4	2	2	0.854 (0.118–6.199)	
AJCC stage, nc					
I	314	162	152	1	1.000b
II	4	2	2	0.938 (0.131–6.744)	

Recurrence/mortality follow-up (months) were described by median or interquartile range. Low recurrence risk, intrathyroidal differentiated thyroid cancer and ≤5 LN micrometastases (<0.2 cm); intermediate recurrence risk, aggressive histology, minor ETE, vascular invasion, or >5 involved lymph nodes (0.2–3 cm); high recurrence risk, gross ETE, incomplete tumor resection, distant metastases or lymph node >3 cm. CI, confidence intervals; CPTC, conventional papillary thyroid cancer; FVPTC, follicular variant papillary thyroid cancer; TCPTC, tall cell variant papillary thyroid cancer; ETE, extrathyroidal extension; RAI, radioactive iodine; T, tumor size; N, lymph node; M, metastasis; AJCC staging, 8th edition American Joint Committee on Cancer staging; aP<0.05; bFisher's exact test; c(11).

**Table II.** Univariate analyses of association between BRAF-V600^E^ mutation status and clinicopathological parameters in ≥55-age group.

		BRAF-V600^E^			
				
Patients' parameters	Total	Mutation	Wild-type	Odds ratio (95% CI)	P-value
Age, years (mean ± standard deviation)	65±9	65±8	65±9	NA	0.976
Sex, n					
Female	103	51	52	1	0.546
Male	54	24	30	0.816 (0.421–1.580)	
Ethnicity category, n					
Caucasian	107	57	50	1	0.428b
Asian	9	5	4	1.096 (0.279–4.309)	
Black	11	3	8	0.329 (0.083–1.308)	
Tumor size, cm (mean ± standard deviation)	1.9±1.0	1.7±0.9	2.1±1.1	NA	0.036a
Tumor foci, n					
Unifocality	85	46	39	1	0.136
Multifocality	69	29	40	0.615 (0.324–1.167)	
Lymphocytic thyroiditis, n					
No	116	59	57	1	0.784
Yes	21	10	11	0.878 (0.346–2.227)	
Histology, n					
CPTC	106	53	53	1	<0.001a
FVPTC	33	7	26	0.269 (0.108–0.674)	
TCPTC	18	15	3	5.000 (1.367–18.287)	
Lymph nodes positivity, n (>5)					
No	93	46	47	1	0.231
Yes	22	14	8	1.788 (0.685–4.665)	
ETE, n (gross)					
No	134	63	71	1	0.169
Yes	17	11	6	2.066 (0.722–5.910)	
Residual tumor, n					
No	110	51	59	1	0.111
Yes	25	16	9	2.057 (0.837–5.051)	
RAI therapy, n					
No	61	35	26	1	0.051
Yes	83	34	49	0.515 (0.264–1.007)	
RAI dose, mCi (mean ± standard deviation)	122±52	125±20	102±62	NA	0.032a
Recurrence follow-up, months	19 (12–34)	21 (12–47)	18 (12-28.3)	NA	0.018a
Recurrence, n					
No	132	58	74	1	0.031a
Yes	15	11	4	3.509 (1.062–11.590)	
Mortality follow-up, months	20 (13–35)	25 (14–48)	18 (12.5–27.5)	NA	0.006a
Mortality, n					
No	143	66	77	1	0.236
Yes	14	9	5	1.968 (0.631–6.136)	
Recurrence risk stage, n					
Low	54	9	45	1	<0.001a
Intermediate	58	42	16	13.125 (5.238–32.887)	
High	39	24	15	8.000 (3.052–20.967)	
T stage, nc					
1	85	32	53	1	0.036a,b
2	37	22	15	2.429 (1.103–5.349)	
3	27	15	12	2.070 (0.861–4.975)	
4	8	6	2	4.969 (0.945–26.116)	
N stage, nc					
0	84	36	48	1	0.036a
1a	18	13	5	3.467 (1.133–10.606)	
1b	26	16	10	2.133 (0.867–5.250)	
M stage, nc					
0	88	44	44	1	1.000b
1	5	3	2	1.500 (0.239–9.420)	
AJCC stage, nc					
I/II	143	64	79	1	0.016a
III/IV	14	11	3	0.938 (0.131–6.744)	

Recurrence/mortality follow-up (months) were described by median or interquartile range. Low recurrence risk, intrathyroidal differentiated thyroid cancer and ≤5 LN micrometastases (<0.2 cm); intermediate recurrence risk, aggressive histology, minor ETE, vascular invasion, or >5 involved lymph nodes (0.2–3 cm); high recurrence risk, gross ETE, incomplete tumor resection, distant metastases or lymph node>3 cm. CPTC, conventional papillary thyroid cancer; CI, confidence intervals; FVPTC, follicular variant papillary thyroid cancer; TCPTC, tall cell variant papillary thyroid cancer; ETE; extrathyroidal extension; RAI, radioactive iodine; T, tumor size; N, lymph node; M, metastasis; AJCC staging, 8th edition American Joint Committee on Cancer staging. aP<0.05; bFisher's exact test; c(11).

**Table III.** Cox multivariate regression analyses of factors associated with recurrence.

	<55-age group		≥55-age group	
		
Clinicopathologic features	HR (95% CI)	P-value	HR (95% CI)	P-value
BRAF	0.842 (0.282–2.513)	0.758	0.995 (0.356–2.781)	0.993
Male sex	1.423 (0.315–6.422)	0.647	2.345 (1.108–4.963)	0.026a
Multifocality	1.753 (0.583–5.272)	0.318	3.180 (2.932–5.850)	0.034a
Histology	0.716 (0.190–2.693)	0.621	1.333 (0.677–2.623)	0.405
ETE	0.716 (0.190–2.693)	0.621	0.422 (0.055–3.256)	0.408
Residual tumor	2.484 (0.682–9.050)	0.168	0.999 (0.219–4.559)	0.999
T stage	1.363 (0.641–2.902)	0.421	1.268 (0.779–2.065)	0.340
N stage	2.453 (1.051–5.722)	0.038a	7.873 (5.121–14.781)	0.001a
M stage	16.010 (1.358–188.707)	0.028a	9.043 (1.966–41.602)	0.005a
AJCC stage	4.215 (0.541–32.855)	0.170	1.093 (0.240–4.968)	0.908

HR, hazard ratios; CI, confidence intervals; ETE, extrathyroidal extension; T, tumor; N, lymph node; M, metastasis; AJCC, the 8th edition American Join Committee on Cancer staging. aP<0.05.

## Data Availability

The datasets generated and/or analyzed during the current study are available in The Cancer Genome Atlas repository, (https://tcga-data.nci.nih.gov).
